# When to be discrete: the importance of time formulation in understanding animal movement

**DOI:** 10.1186/s40462-014-0021-6

**Published:** 2014-10-15

**Authors:** Brett T McClintock, Devin S Johnson, Mevin B Hooten, Jay M Ver Hoef, Juan M Morales

**Affiliations:** National Marine Mammal Laboratory, NOAA-NMFS Alaska Fisheries Science Center, Seattle, WA 98115 USA; U.S. Geological Survey, Colorado Cooperative Fish and Wildlife Research Unit, Department of Fish, Wildlife, and Conservation Biology, Department of Statistics, Colorado State University, Fort Collins, CO 80523 USA; National Marine Mammal Laboratory, NOAA-NMFS Alaska Fisheries Science Center, Fairbanks, AK 99775 USA; Ecotono, INIBIOMA–CONICET, Universidad Nacional del Comahue, Quintral 1250, Bariloche, 8400 Argentina

**Keywords:** Animal location data, Diffusion, Movement model, Random walk, State-space model, Switching behavior, Telemetry

## Abstract

Animal movement is essential to our understanding of population dynamics, animal behavior, and the impacts of global change. Coupled with high-resolution biotelemetry data, exciting new inferences about animal movement have been facilitated by various specifications of contemporary models. These approaches differ, but most share common themes. One key distinction is whether the underlying movement process is conceptualized in discrete or continuous time. This is perhaps the greatest source of confusion among practitioners, both in terms of implementation and biological interpretation. In general, animal movement occurs in continuous time but we observe it at fixed discrete-time intervals. Thus, continuous time is conceptually and theoretically appealing, but in practice it is perhaps more intuitive to interpret movement in discrete intervals. With an emphasis on state-space models, we explore the differences and similarities between continuous and discrete versions of mechanistic movement models, establish some common terminology, and indicate under which circumstances one form might be preferred over another. Counter to the overly simplistic view that discrete- and continuous-time conceptualizations are merely different means to the same end, we present novel mathematical results revealing hitherto unappreciated consequences of model formulation on inferences about animal movement. Notably, the speed and direction of movement are intrinsically linked in current continuous-time random walk formulations, and this can have important implications when interpreting animal behavior. We illustrate these concepts in the context of state-space models with multiple movement behavior states using northern fur seal (*Callorhinus ursinus*) biotelemetry data.

## Introduction

Animal movement is at the heart of many important ecological processes and considered essential for a better understanding of population dynamics, animal behavior, and the impacts of global change. However, movement is a complex process modulated by many factors acting at different spatial and temporal scales. Our ability to study animal movement has been bolstered by recent advances in animal-borne biologging technology that have permitted the collection of detailed location and biotelemetry data [1-3]. The quality and quantity of information from these devices is rapidly increasing, and there has been a recent flood in the development of sophisticated statistical models that use these data for model-based inferences about animal movement and associated behaviors [4-8].

This myriad of new methods for analyzing movement data can make the selection of any particular method (or model) a difficult task, particularly for ecologists and wildlife biologists without formal statistical training. This poses a dilemma because ecologists and biologists constitute the vast majority of scientists collecting the very data for which these methods were developed. The complexities of animal movement and location data require sophisticated analytical techniques, but we believe that the inconsistent mathematical and statistical jargon used to describe these methods may be discouraging their widespread application by non-statisticians. In our experience, the greatest source of confusion among practitioners, both in terms of implementation and biological interpretation, seems to be the distinction between continuous- and discrete-time formulations of the movement process.

Here we briefly review several of the model-based (non-phenomenological) approaches for analyzing animal location data that have been proposed in recent years. We then focus on how time is formulated in these movement process models, establish some common terminology (see Table 1), elucidate the differences and similarities among them, and identify some potential advantages and limitations. We also present novel mathematical results (see **Does a continuous- or discrete-time formulation really matter?**) refuting the overly simplistic view that discrete- and continuous-time conceptualizations are merely different means to the same end in terms of inferences about animal movement. We then illustrate these concepts in the context of state-space models with multiple movement behavior states using northern fur seal (*Callorhinus ursinus*) movement data collected in the Pribilof Islands of Alaska, USA.

**Table 1:** **Glossary**

**Term**	**Definition**	**Synonyms**
Behavioral state	A discrete (and typically latent) behavior associated with a specific type of movement.	Behavior; behavioral mode
Brownian motion	A simple random walk in continuous time, i.e., a diffusion model with no centralizing tendency.	Wiener process
Central tendency	A tendency to move back towards a central location (e.g., the center of a home range or patch) as a result of directed movement.	Mean-reverting
Correlated movement	Short-term directional persistence resulting from a tendency to continue moving in a similar direction (or velocity) as previous moves.	
Directed movement	Systematic, non-random movement in a particular direction. Directed movement associated with a particular location or gradient, such as a “center of attraction,” can result in long-term directional persistence and/or central tendency.	Biased or oriented movement (discrete time); drift or advection (continuous time)
Directional persistence	A tendency for successive movements to be in a similar direction.	
Hidden Markov model	A special class of state-space models with a finite number of hidden (e.g., behavioral) states.	
Markov process	A stochastic process where state transitions are dependent only on the current state (first-order Markov process) or current and immediately previous states (higher-order Markov process).	
Multistate model	A mixture of random walk models corresponding to different movement behavior states.	Mixture model, switching model
Ornstein-Uhlenbeck (OU) process	A diffusion model with centralizing tendency that accounts for dependence between observations. With no central tendency, Brownian motion is obtained as a limiting case.	
Random walk	Given an initial starting position, a mathematical model for generating a stochastic movement trajectory in space. Random walks are often Markov processes and can be formulated in discrete or continuous time. They have no directional persistence or bias.	
State-space model	A conditionally specified hierarchical model consisting of a latent system process model and an observation model.	

## Review

### Characterization of the movement process

Regardless of the underlying statistical framework, most analyses of animal location data that are based on hierarchical movement models consist of two components: a mechanistic model for the movement process and a statistical model for the observation process. Although earlier methods ignored error in the location of observations [5,9,10], most contemporary approaches simultaneously model both the movement process and observation process using a so-called’ “state-space” framework [6,8,11,12].

Recent technological advances (e.g., GPS) are making location measurement error less of a concern, and this has allowed greater focus on the development of more realistic (and biologically meaningful) models for the movement process. These developments primarily differ in the spatio-temporal conceptualization of the movement process, including discrete-time and discrete-space [13-15], discrete-time and continuous-space [5,6], continuous-time and discrete-space [16,17], and continuous-time and continuous-space [8,9] movement process models (see Table 2). Although time formulation in continuous space is our primary focus henceforth, discrete-space movement models are often employed in the absence of detailed location data (e.g., capture-mark-recapture studies e.g., [14,16]), or resource selection studies in heterogeneous environments e.g., [17]. Latent behaviors associated with different types of movement can also be treated as continuous [18] or discrete [5,6,19,20] states among which individuals transition in response to changes in their internal and external environment. Other approaches go a step further by attempting to combine “macroscopic” resource selection models with “microscopic” discrete- or continuous-time movement process models [7,21-27].

**Table 2:** **Summary of conventional mechanistic movement process models based on spatiotemporal formulation (time and space), movement metric, types of movement that are accounted for (directed or correlated), and accommodation of multiple movement behavior states using multistate models**

**Time**	**Space**	**Metric**	**Directed**	**Correlated**	**Multistate**	**Reference**
discrete	discrete	position	NA	NA	yes	[13-15]
discrete	continuous	position	yes	no	yes	[28]
discrete	continuous	position	yes	no	no	[7]
discrete	continuous	velocity	no	yes	yes	[6]
discrete	continuous	step length	no	yes	yes	[18]
discrete	continuous	step length and turning angle	no	yes	yes	[5]
discrete	continuous	step length and bearing	yes	no	no	[29]
discrete	continuous	step length and bearing	yes	yes	yes	[20,30]
continuous	discrete	position	yes	no	yes	[16]
continuous	discrete	position	yes	yes	no	[17]
continuous	discrete	velocity	yes	yes	yes	[19]
continuous	continuous	position	yes	no	no	[10]
continuous	continuous	position	yes	no	yes	[4,9,31]
continuous	continuous	step length and turning angle velocity	no	yes	yes	[32]
continuous	continuous	velocity	no	yes	no	[33]
continuous	continuous	velocity	yes	yes	no	[8]
continuous	continuous	velocity	yes	yes	yes	[31]

Example references are also provided.

Before proceeding, we note that hierarchical discrete-time, continuous-space movement process models are often referred to as “state-space” models in the literature. This is not a misnomer. However, based on conventional time series jargon, any approach that simultaneously accounts for the system process (i.e., the movement process) and the observation process through time qualifies as a state-space model. In this sense, all of the hierarchical modeling approaches above employ state-space methods. In the contemporary statistical literature, state-space models are now more commonly referred to as hierarchical models; “hierarchical” because the data arise from a probability distribution that depends on a latent process, which, in turn, is modeled stochastically [34,35]. We also note that discrete-time movement models where each behavioral state is associated with a distinct random walk [5,6,20,30] can be considered as hidden Markov models, a special class of state-space models with a finite number of latent states [36].

In general, animal movement occurs in continuous time but we observe it at fixed discrete-time intervals. Thus, continuous-time models are conceptually and theoretically appealing, but in practice it is perhaps more intuitive to interpret movement in discrete intervals (e.g., turning angle and step length per unit time). It is easier to conceptualize the movement process as a series of steps and turns sampled from particular distributions than to deal with partial differential equations. This may in part explain why the methodological development and application of discrete-time models has thus far exceeded that of continuous-time models.

Whether in discrete or continuous time, most mechanistic movement process models are based on correlated random walks. In discrete time, correlated movement is typically modeled with non-uniform turning angle distributions, usually with mean of zero, which result in short-term directional persistence between successive time steps. The more highly correlated movement exhibits turning angles tending towards zero [5,6]. In continuous time, correlated movement can be expressed through a special type of diffusion model that accounts for dependence between locations, the Ornstein-Uhlenbeck (OU) process [4,10]. The OU process is essentially a continuous-time random walk with a tendency to drift towards a central location. Using an OU process to model movement velocity instead of locations, Johnson et al. [8] developed a correlated random walk model that is a continuous-time analog to the discrete-time model of Jonsen et al. [6].

Both discrete- and continuous-time random walk models can incorporate directed (or oriented) movement, but this is often referred to as “biased” movement in discrete-time models [20,37] and “drift” or “advection” in continuous-time models [4,10]. Directed movements are typically associated with specific locations in space, such as “centers of attraction” or “centers of repulsion,” and can be used to model a general tendency towards the center of a home range [7,10] or patch [4,20,31]. Thus, directional persistence can result from directed movements, but the long-term directional persistence that can result from directed movement is different from the short-term directional persistence associated with a correlated random walk [38]. Under directed movement, longer-term directional persistence results from an individual being constantly pulled towards (or pushed away from) a particular location or gradient (without explicit consideration of the direction of previous movements).

Without correlated movements, the discrete-time models of Morales et al. [5] and Jonsen et al. [6] reduce to simple random walks. Without directed movements, the discrete-time model of McClintock et al. [20] reduces to the correlated random walk model of Morales et al. [5]. The OU process models of Dunn and Gipson [10], Blackwell [4,9], Johnson et al. [8], and Harris and Blackwell [31] reduce to Brownian motion (i.e., a continuous-time simple random walk), using a mathematical limit argument. We note that because the directional persistence in a correlated random walk decays exponentially as the time lapse increases, correlated random walks can be approximated at larger scales with a simple diffusion model [16].

To incorporate both correlated and directed movement, the expected direction of movement must reflect a trade-off between short-term directional persistence and the strength of bias towards (or away from) a center of attraction (or repulsion). This has been examined in discrete time by modeling the expected direction as a weighted average of the strength of bias in the direction of the center of attraction and the previous movement direction [20,37]. Although a similar approach has yet to be thoroughly investigated in continuous time, this would be akin to modeling the drift parameter of an OU process as a function of both directed and correlated movements.

### The metrics of movement

Movement metrics also differ among the aforementioned approaches by specifying the movement process on the positions themselves [7,9,28] or on derived quantities, such as the differences between consecutive locations (i.e., velocities) [6,8,19,32,33], step lengths [18], step lengths and turning angles [5], or step lengths and bearings [20,29] (see Table 2). These movement metrics are important for model specification and interpretation. For example, by modeling velocity, the discrete-time model of Jonsen et al. [6] and the continuous-time model of Johnson et al. [8] induce dependence between the speed and direction of movement, so that long steps are possible when turning angles are small, resulting in higher-order auto-correlations than found in standard correlated random walks [5,20]. Although Blackwell [4,9] models position and Johnson et al. [8] model velocity, the speed and direction of movement are intrinsically linked through the drift process of these continuous-time models (see **Does a continuous- or discrete-time formulation really matter?**). By modeling turning angles independent of step lengths in discrete-time, Morales et al. [5] could investigate correlated (but not directed) movements independent of speed. By modeling bearings using a similar discrete-time movement process model, McClintock et al. [20] could simultaneously investigate both correlated and directed movements independent of speed.

### Does a continuous- or discrete-time formulation really matter?

Outside of fitting them to data and empirically assessing differences, it is not immediately apparent how alternative time formulations of movement models differ analytically. In fact, continuous- and discrete-time formulations are often over simplistically viewed as merely different means to the same end. But this is not the case, and we derive a partial translation here to compare continuous- and discrete-time formulations with a common and intuitive language: step length and bearing.

Kobayashi et al. [39] provides the following necessary result for two independent normally-distributed random variables, *A* and *B*. If

$$ \left[A,B\right]=\mathcal{N}\left({\mu}_A,{\sigma}^2\right)\mathcal{N}\left({\mu}_B,{\sigma}^2\right), $$

then the distance from the origin, $L=\sqrt{A^2+{B}^2},$ has a Rice distribution, *R*(*μ*, *σ*^2^), where $\mu =\sqrt{\mu_A^2+{\mu}_B^2}=\left\Vert \boldsymbol{\mu} \right\Vert$ is the distance from the origin to the center of the bivariate normal distribution, *σ*^2^ is a variance parameter, and $\mathcal{N}\left(\right)$ is the Normal probability density function. The Rice distribution is a generalization of the Rayleigh distribution (for *μ* ≠ 0) whose expected value increases with increasing values of *μ*. Further, the bearing *θ* = tan^− 1^(*B*/*A*), has the conditional von Mises distribution

$$ \left[\theta \left|L=l\right.\right]=VM\left(\omega, \kappa \right)=\frac{{\mathrm{e}}^{\kappa \cos \left(\theta -\omega \right)}}{2\pi {I}_0\left(\kappa \right)}, $$

where *κ* = *lμ*/*σ*^2^, *ω* = tan^− 1^(*μ*_*B*_/*μ*_*A*_), and *I*_0_() is the modified Bessel function of the first kind and of order 0. The von Mises distribution is symmetric and centered on the angle *ω*, and dispersion decreases with increasing *κ* values.

We can now translate a time step of the continuous-time correlated random walk (CTCRW) model of Johnson et al. [8] to a discrete-time step length and bearing. First, the transformation of the bivariate velocity process to speed (distance unit per time unit) and direction is given by

$$ {l}_t=\left|\right|{v}_t\left|\right| $$

and

$$ {\theta}_t={ \tan}^{-1}\left({V}_{y,t}/{V}_{x,t}\right). $$

The resulting distributions are obtained by applying the results in Kobayashi et al. [39] to the CTCRW velocity model equations (see Eqs. 3 and 4 in ***Continuous-time formulation*** below). Using the velocity process transformation, the location step length ${S}_t=\sqrt{{\left({X}_{t+1}-{X}_t\right)}^2+{\left({Y}_{t+1}-{Y}_t\right)}^2}$ and bearing *ϕ*_*t*_ = tan^− 1^{(*Y*_*t* + 1_ − *Y*_*t*_)/(*X*_*t* + 1_ − *X*_*t*_)} are distributed as

$$ \begin{array}{l}\left[{S}_t\mid {l}_t,{Z}_t=z\right]=R\left({l}_t\left(1-{e}^{-{\beta}_z}\right)/{\beta}_z,{q}_{z,t}\right)\hfill \\ {}\left[{\phi}_t\mid {S}_t,{l}_t,{Z}_t=z\right]=VM\left({\theta}_t,{S}_t{l}_t{e}^{-{\beta}_z}/{q}_{z,t}\right),\hfill \end{array} $$

where *Z*_*t*_ is the latent behavioral state, and *q*_*z*,*t*_ is the (1,1) (or, (2,2) as they are the same) entry of the covariance matrix for the velocity process (**Q**_*z*,*t*_).

There are now notable differences that one can easily distinguish between the continuous and discrete formulations for step length and bearing distributions. First, unlike the discrete-time model (see Eqs. 1 and 2 in ***Discrete-time formulation*** below), the step length and bearing of the continuous-time model are clearly correlated. As step length increases the distribution of the bearing becomes more concentrated around *θ*_*t*_, the latent velocity bearing. Second, given a constant state process, step lengths are independent in the discrete-time formulation. However, in the CTCRW model step lengths are still correlated via the auto-correlated speed process, *l*_*t*_. Thus, unlike the discrete-time model, the CTCRW maintains not just directional persistence, but persistence in speed as well. Note that this result does not depend on latent behavioral state (*Z*_*t*_) and holds for movement models with a single behavioral state.

We emphasize that these results are not simply attributable to the fact that the CTCRW model is based on an integrated OU velocity. They hold analogously for continuous-time models which use OU process models for position directly [4,9,31], even if *X*_*t*_ and *Y*_*t*_ are modeled independently (i.e., by setting the off-diagonal elements of the covariance matrix for the bivariate OU process to zero). Using the same result from Kobayashi et al. [39], the distributions of the step length and bearing of an OU process directly modeling position, with central location ***μ*** = (*μ*_*x*_, *μ*_*y*_), are

$$ \begin{array}{l}\left[{S}_t\mid {Z}_t=z\right]=R\left({D}_t\left(\boldsymbol{\mu} \right)\left\{{e}^{-{\beta}_z}-1\right\},{\sigma}_t^2\right)\hfill \\ {}\left[{\phi}_t\mid {S}_t,{Z}_t=z\right]=VM\left({\theta}_t,{S}_t\left\{{e}^{-{\beta}_z}-1\right\}\right)\hfill \end{array} $$

where *D*_*t*_(***μ***) and *θ*_*t*_ are respectively the distance and bearing from the current position to the central location, and ${\sigma}_t^2$ is the variance of the OU process at time *t*. One can see that the OU model directly applied to the positions still maintains correlation between step length and bearing. Moreover, it also possesses the (potentially undesirable) quality that movement rate depends on distance from the point of attraction, thus necessitating rapid movement that slows as the animal approaches the central location.

### Potential advantages and disadvantages

Given the various ways by which similar movement properties can be expressed using either discrete- or continuous-time process models, some potential advantages and disadvantages are evident. Although animal movement clearly occurs in continuous time, discrete-time models are often viewed as more intuitive, and perhaps the biological interpretation of instantaneous movement parameters in continuous time (e.g., those related to OU processes and other diffusion models) can in practice be discouraging to applied ecologists wishing to use or extend continuous-time methods.

Notably, discrete-time models that simultaneously incorporate multiple latent movement behavior states, Markov state-switching, correlated movements, and directed movements have already been developed and fitted to data [5,6,20]. For example, Morales et al. [5] used a discrete-time random walk mixture model to examine time allocations and transition probabilities between two latent movement behaviors in elk: a long-step, directionally-persistent “exploratory” state and a short-step, negatively-correlated (i.e., with animals tending to move in the opposite direction of the previous move) “encamped” state. Similarly, Jonsen et al. [6] investigated analogous “transit” and “foraging” movement behavior states in seals. Also using seal data, McClintock et al. [20] developed a biased, correlated random walk mixture model with five latent movement behavior states allowing for directed and exploratory movement among foraging and haul-out locations.

Similar applications of multistate mixture models have yet to appear in continuous-time (but see **Example: northern fur seal**). Blackwell [9] assumed movement behavior states were known, and Johnson et al. [8] assumed states were defined by known covariates, hence neither of these approaches included an estimation framework for both latent movement states and switching behavior. Hanks et al. [19] extended the framework of Johnson et al. [8] and Hooten et al. [17] to accommodate inhomogeneous movement characteristics along the movement path using a change-point model. However, because this approach does not explicitly incorporate distinct movement behavior states or state-switching mechanisms with direct biological interpretation, *post hoc* cluster analyses were used to identify potential movement behavior states. Harris and Blackwell [31] recently described a continuous-time multistate mixture modeling framework, but fitting these models is challenging, and they have yet to be demonstrated using real data. Part of the difficulty of multistate mixture models in continuous time is due to the underlying relationships these models typically impose on the movement characteristics (e.g., speed or directional persistence) commonly used to distinguish movement behavior states (see **Does a continuous- or discrete-time formulation really matter?** and **Example: northern fur seal**). Because multistate models are of great practical importance for investigating time allocations to different behaviors (i.e., “activity budgets”), this currently remains an advantage of discrete-time models.

Two important disadvantages of discrete-time models are related to the necessary discretization of the movement path into a finite number of temporally-regular time steps [40]. The time step length must be specified *a priori*, but inferences about animal movement from a discrete-time analysis are not time scale-invariant. For example, inferences about bumblebee movement characteristics from discrete-time analyses using 30-second versus 30-minute time steps would likely be dramatically different. The 30-second analysis would reveal fine-grain movement properties but could potentially mask coarser-grain properties. The 30-minute analysis could reveal coarse-grain properties, but would completely miss fine-grain properties. The specification of time step length in a discrete-time analysis is therefore critical and requires very careful consideration [41-43], and it is particularly important that the time step is chosen to match the scale at which behavioral decisions are made [40]. A major advantage of continuous-time models is that they avoid dependence on a particular timescale. Within reasonable limits, a continuous-time analysis will yield the same results regardless of the temporal resolution of observations; if so desired, movement properties from a continuous-time analysis may be summarized *a posteriori* for time steps of any length. However, we note that for any continuous- or discrete-time approach to be useful, the temporal resolution of the observed data must be relevant to the specific movement behaviors of interest.

Discrete-time movement models can also be more computationally demanding than continuous-time models. Unless observations exactly match the regular time steps required of a discrete-time model, the movement path must be predicted at temporally-regular intervals. Perfectly observed, temporally-regular observations are very rare in animal telemetry data (especially for marine species). For longer time series, this can result in thousands of additional location parameters that must be estimated. As movement process models incorporate more details and realism, model fitting becomes more complex. This is particularly true for multistate mixture models. Therefore, once multistate model development and fitting in continuous time has caught up with that in discrete time, the computational advantages of continuous-time formulations are likely to be significant.

### Example: northern fur seal

To illustrate the concepts elaborated above in the context of state-space models with latent movement behavior states, we apply comparable multistate movement models in discrete and continuous time to a northern fur seal track in the Pribilof Islands of Alaska, USA. The animal was a nursing female equipped with a Mk10-AF satellite tag from Wildlife Computers (see [44] for full study deployment details). The Mk10-AF tag has both Fastloc GPS and time-depth recording capabilities. Using both location and diving activity data, we wish to identify and characterize three latent movement behavior states: “resting,” “foraging,” and “transit”. We define foraging (state F) as movement that is characteristic of area restricted searches and includes foraging dives, where a foraging dive must have a max depth >5 m and at least 5 changes in vertical direction (i.e., sinuosities or “wiggles”). The sinuosities are a characteristic of the animal chasing prey during the dive. We define transit (state T) as predominantly travelling with little to no foraging dives, noting that seals may opportunistically feed while travelling. Resting (state R) is defined by types of movement that do not fall under foraging or transit states, including resting at haulouts and resting at sea. In terms of trajectory, we would expect speeds to be low during resting and low to moderate during foraging, with little directional persistence. During transit, we would expect higher speeds and greater directional persistence.

**Figure 1:**
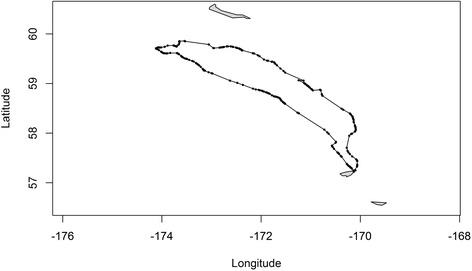
**Observed locations during a foraging trip 10–17 October 2007 for a northern fur seal that hauls out in the Pribilof Islands, Alaska.**

The diving activity data were summarized as the number of foraging dives for each of *N* = 242 1-hour time steps between 7–17 October 2007. Although diving data were logged continuously, location data were obtained opportunistically at 15-minute intervals. There are therefore frequent missing location data due to an inability to obtain locations while the seal was underwater. Because the tag possessed GPS capabilities, rather than ARGOS technology, we expect location measurement error to be minimal. The raw location data consist of 241 observations during a single foraging trip (Figure 1), with 40% of the 1-hour time steps containing no observed locations.

#### Discrete-time formulation

With the location data being temporally irregular, a discrete-time analysis requires that the movement path be estimated at regular time steps. We chose 1-hour time steps to exactly match the temporal resolution of the foraging dive data. Using the same state-space formulation as McClintock et al. [20], for time step *t* = 1, …, *N*, and observation *i* = 1, …, *k*_*t*_, we relate the irregularly observed locations (*x*_*t*,*i*_, *y*_*t*,*i*_) to the temporally regular model locations (*X*_*t*_, *Y*_*t*_) using

$$ {x}_{t,i}=\left(1-{j}_{t,i}\right){X}_{t-1}+{j}_{t,i}{X}_t+{\epsilon}_{x_{t,i}}, $$

and

$$ {y}_{t,i}=\left(1-{j}_{t,i}\right){Y}_{t-1}+{j}_{t,i}{Y}_t+{\epsilon}_{y_{t,i}}, $$

where *j*_*t*,*i*_ ∈ [0, 1) is the proportion of the time interval between locations (*X*_*t* − 1_, *Y*_*t* − 1_) and (*X*_*t*_, *Y*_*t*_) at which the *i*^th^ observation between times *t-* 1and *t* was obtained, $$ \left[{\epsilon}_{x_{t,i}}\right]=\mathcal{N}\left(0,{\sigma}_x^2\right), $$$$ \left[{\epsilon}_{y_{t,i}}\right]=\mathcal{N}\left(0,{\sigma}_y^2\right),\kern0.5em \left[\dots \right] $$ indicates the probability density function for the random variable in brackets, and $$ \mathcal{N}\left(\right) $$ is the Normal (Gaussian) density. Time steps with no observations (i.e., *k*_*t*_ = 0) do not contribute to the observation model.

We then model movement between the temporally regular locations using a multistate correlated random walk model [5,20]. Specifically, we assume that, conditional on the behavioral state, *Z*_*t*_, the step length at time *t*, *S*_*t*_, is distributed as

1 $$ \left[{S}_t\Big|\boldsymbol{a},\boldsymbol{b},{Z}_t=z\right]= Weibull\left({a}_z,\ {b}_z\right) = \frac{b_z}{a_z}{\left(\frac{S_t}{a_z}\right)}^{b_z-1}\times \exp \left[-{\left(\frac{S_t}{a_z}\right)}^{b_z}\right] $$

where *S*_*t*_ ≥ 0, *z* ∈ {*R*, *F*, *T*} is the unknown latent behavioral state, and *a*_*z*_ and *b*_*z*_ are state-dependent scale and shape parameters, respectively. The Weibull distribution is popular for modeling step length because of its flexibility; it has fat tails when *b*_*z*_ < 1, reduces to an exponential distribution when *b*_*z*_ = 1, has exponential tails when *b*_*z*_ > 1, and can resemble a normal distribution when *b*_*z*_ ≈ 3.4. The bearing of movement, *ϕ*_*t*_, is modeled with the wrapped Cauchy distribution

2 $$ \left[{\phi}_t\Big|{\phi}_{t-1},\boldsymbol{\rho}, {Z}_t=z\right]= wCauchy\left({\phi}_{t-1},\ {\rho}_z\right)=\frac{1-{\rho}_z^2}{2\pi \left[1+{\rho}_z^2-2{\rho}_z \cos \left({\phi}_t-{\phi}_{t-1}\right)\right]} $$

where 0 ≤ *ϕ*_*t*_ < 2*π*, *ϕ*_*t* − 1_ is the previous bearing, and − 1 < *ρ*_*z*_ < 1 is the state-dependent dispersion parameter. Unfamiliar to most non-statisticians, the wrapped Cauchy distribution converges to a uniform distribution over the circle as *ρ*_*z*_ goes to zero. As *ρ*_*z*_ goes to 1 (or − 1), the distribution tends to a point mass concentrated towards (or away from) the previous bearing. Standard correlated movement is typically modeled with the wrapped Cauchy distribution by constraining 0 ≤ *ρ*_*z*_ < 1 [5,45].

It can be difficult to distinguish resting, foraging, and transit states for seals based on trajectory alone [45], particularly because northern fur seals can forage opportunistically while travelling and will often rest at sea or in the vicinity of breeding rookeries. We therefore incorporate the number of foraging dives during each time step, *δ*_*t*_, to help inform the foraging state. Specifically, we assume

$$ \left[{\delta}_t\Big|\boldsymbol{\lambda}, {Z}_t=z\right]= Poisson\left({\lambda}_z\right) $$

with the constraints *λ*_*R*_ = 0 and *λ*_*F*_ > *λ*_*T*_. This model therefore assumes *a priori* that time steps with foraging dives are never assigned to resting, and steps with relatively many foraging dives are more likely to be assigned to foraging than transit. Note that by constraining *λ*_*F*_ > *λ*_*T*_, we still allow some possibility for steps with foraging dives to be assigned to transit.

Finally, we model switches between behavior states as a first-order Markov process. We assign the conditional distribution to the latent state variable *Z*_*t*_

$$ \left[{Z}_t\Big|\boldsymbol{\psi}, {Z}_{t-1}=z\right]= Categorical\left({\psi}_{z,R},\ {\psi}_{z,F},{\psi}_{z,T}\right) $$

where for $z,\ {z}^{\prime}\in \left\{R,F,T\right\},{\psi}_{z,{z}^{\prime }}$ is the probability of switching from state *z* at time *t* – 1 to state *z*′ at time *t*.

Using Bayesian analysis methods, the joint posterior distribution for our state-space model in discrete time is

$$ \begin{array}{l}\left[\boldsymbol{a},\boldsymbol{b},\boldsymbol{\rho}, \boldsymbol{\lambda}, \boldsymbol{\psi}, {\sigma}_x^2,{\sigma}_y^2,{X}_0,{Y}_0,\boldsymbol{\phi}, \mathbf{S},\mathbf{Z}\left|\mathbf{x},\mathbf{y},\boldsymbol{\updelta} \right.\right]\hfill \\ {}\propto {\displaystyle \prod_{t=1}^N\left\{\left[{S}_t\left|\boldsymbol{a},\boldsymbol{b},{Z}_t\right.\right]\left[{\phi}_t\left|{\phi}_{t-1},\boldsymbol{\rho}, {Z}_t\right.\right]\left[{\delta}_t\left|\boldsymbol{\uplambda}, {Z}_t\right.\right]\left[{Z}_t\Big|\boldsymbol{\psi}, {Z}_{t-1}\right]\right.}\hfill \\ {}\times {\displaystyle \prod_{i=1}^{k_t}\left.\left[{x}_{t,i},{y}_{t,i}\left|{\sigma}_x^2,{\sigma}_y^2\right.{X}_0,{Y}_0,{\boldsymbol{\phi}}_{\left[1:t\right]},{\mathbf{S}}_{\left[1:t\right]}\right]\right\}}\times \left[\boldsymbol{a}\right]\left[\boldsymbol{b}\right]\left[\boldsymbol{\rho} \right]\left[\boldsymbol{\psi} \right]\left[\boldsymbol{\lambda} \right]\left[{\sigma}_x^2\right]\left[{\sigma}_y^2\right]\left[{X}_0,{Y}_0\right]\hfill \end{array} $$

where (*X*_0_, *Y*_0_) is the initial (latent) location. Note that, conditional on *Z*_*t*_, this discrete-time model assumes step length, bearing, and the number of foraging dives are independent. Weakly informative priors were used for all parameters, including the conjugate priors $$ \left[{\sigma}_x^2\right]={\Gamma}^{-1}\left(0.01,0.01\right), $$$$ \left[{\sigma}_y^2\right]={\Gamma}^{-1}\left(0.01,0.01\right), $$ [*λ*_*z*_] = Γ(0.01, 0.01) for *z* ∈ {*F*, *T*}, and [***ψ***_*z*_] = *Dirichlet*(1, 1, 1) for *z* ∈ {*R*, *F*, *T*}, where Γ() and Γ^− 1^() are the gamma and inverse gamma probability density functions, respectively. For [*X*_0_, *Y*_0_], we specified a joint uniform prior over the region defined by the Bering Sea. We specified a maximum sustainable speed of 3 m/s, such that *S*_*t*_ ≤ 10800m, with [*a*_*z*_] = *Unif*(0, 10800), [*b*_*z*_] = *Unif*(0, 30), and [*ρ*_*z*_] = *Unif*(0, 1) for *z* ∈ {*R*, *F*, *T*}. Similar to McClintock et al. [20,45], we used a Metropolis-within-Gibbs Markov chain Monte Carlo algorithm written in the C programming language [46] to obtain samples from the posterior distribution, performing pre- and post-processing in R via the .C interface [47]. The only notable difference from the MCMC algorithm for the individual-level model of McClintock et al. [45] results from our model for *δ*_*t*_, for which the conjugate prior on *λ*_*z*_ yields the full conditional distributions $$ \left[{\lambda}_F\left|\cdot \right.\right]={\Gamma}_{\left({\lambda}_T,\infty \right)}\left(0.01+{\displaystyle {\sum}_{t=1}^N{\delta}_t{I}_{\left\{{Z}_t=F\right\}},}0.01+{\displaystyle {\sum}_{t=1}^N{I}_{\left\{{Z}_t=F\right\}}}\right) $$ and $$ \left[{\lambda}_T\left|\cdot \right.\right]={\Gamma}_{\left(0,{\lambda}_F\right)}\left(0.01+{\displaystyle {\sum}_{t=1}^N{\delta}_t{I}_{\left\{{Z}_t=T\right\}},}0.01+{\displaystyle {\sum}_{t=1}^N{I}_{{}_{\left\{{Z}_t=T\right\}}}}\right), $$ where Γ_(*l*,*u*)_ is the renormalized gamma density truncated at *l* and *u*, 0 ≤ *l* < *u*, and *I*() is the indicator function. When full conditional distributions were analytically intractable, random walk Metropolis-Hastings parameter updates were used. After initial pilot tuning and burn-in, a single chain of 5 million iterations was attained for posterior summaries. The algorithm required approximately 3 hours to run on a machine running 64-bit Windows 7 (3.4GHz Intel Core i7 processor, 16Gb RAM).

**Figure 2:**
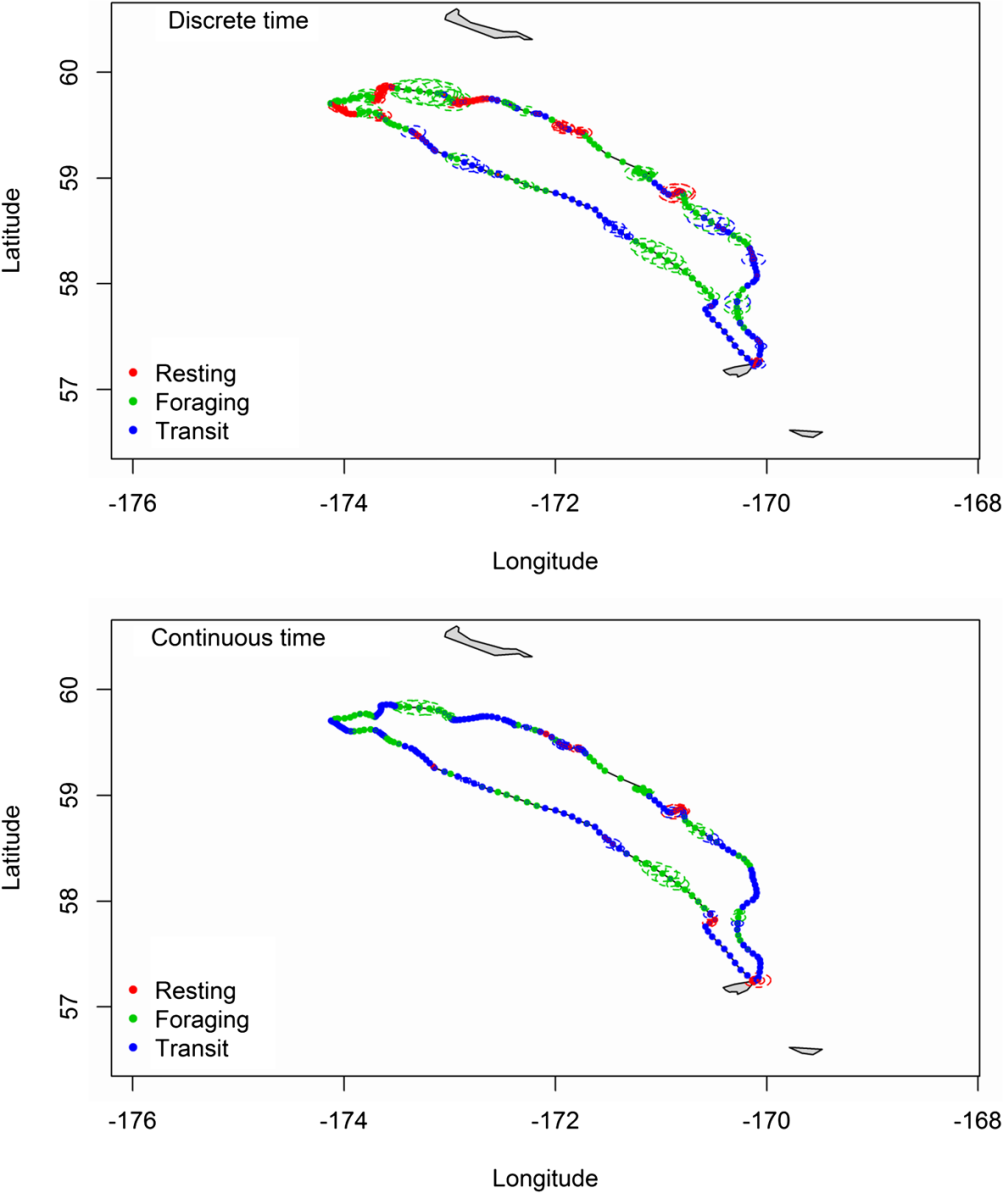
**Estimated path and movement behavior states during a foraging trip of a northern fur seal that hauls out in the Pribilof Islands, Alaska.** Results are presented for discrete- and continuous-time movement process models. Estimated movement states for the predicted locations correspond to “resting” (red), “foraging” (green), and “transit” (blue) movement behavior states. Uncertainty in the state assignments (<95% posterior probability) are indicated by hollow circles within predicted locations. Uncertainty in predicted locations are indicated by 95% credible bands (dashed lines).

**Figure 3:**
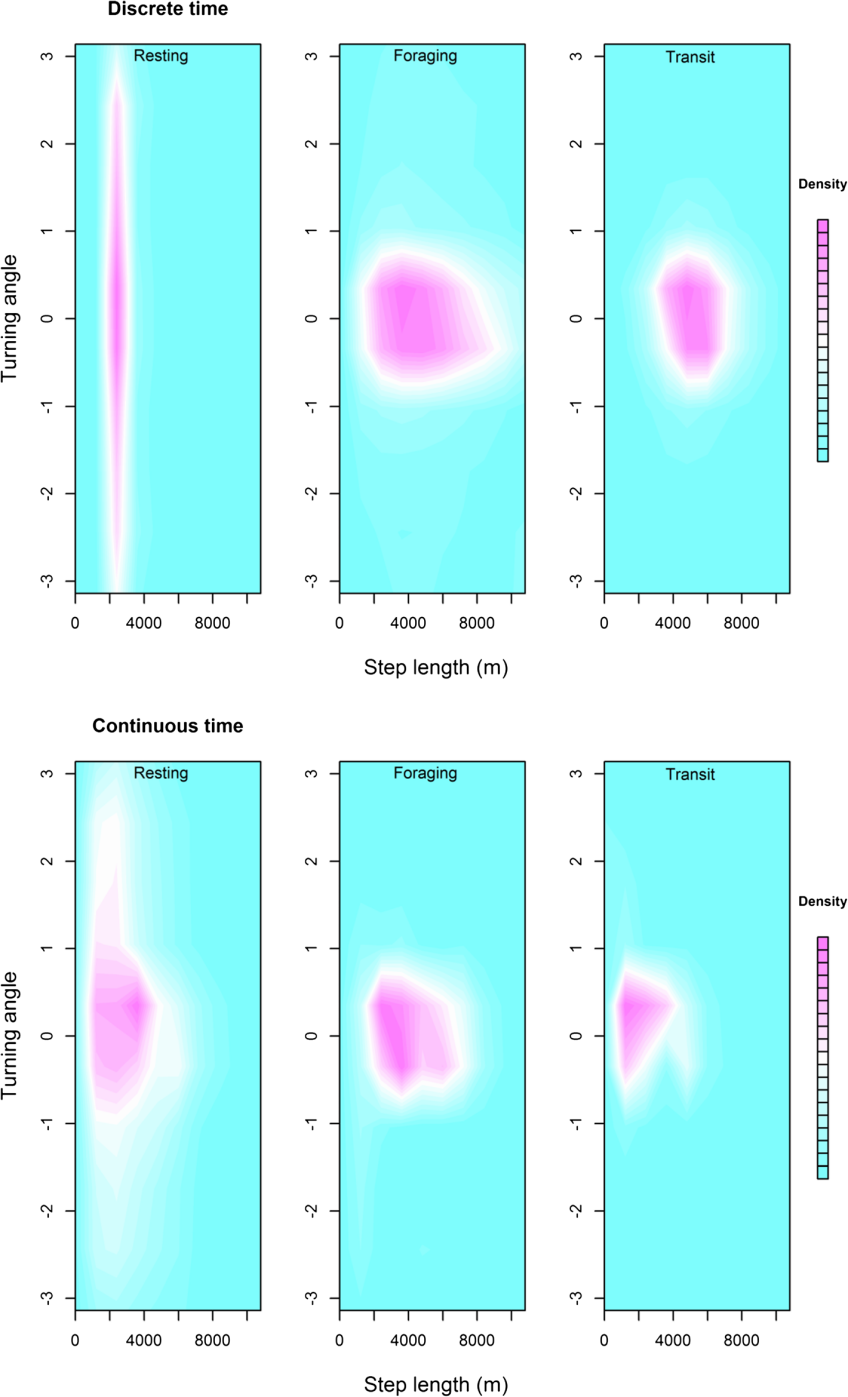
**Estimated bivariate densities of northern fur seal step lengths and turning angles for three distinct movement behavior states (“resting”, “foraging”, and “transit”) based on discrete- and continuous-time movement process models with 1-hour time steps.** For both models, step lengths and turning angles were calculated from the estimated paths shown in Figure 2.

**Figure 4:**
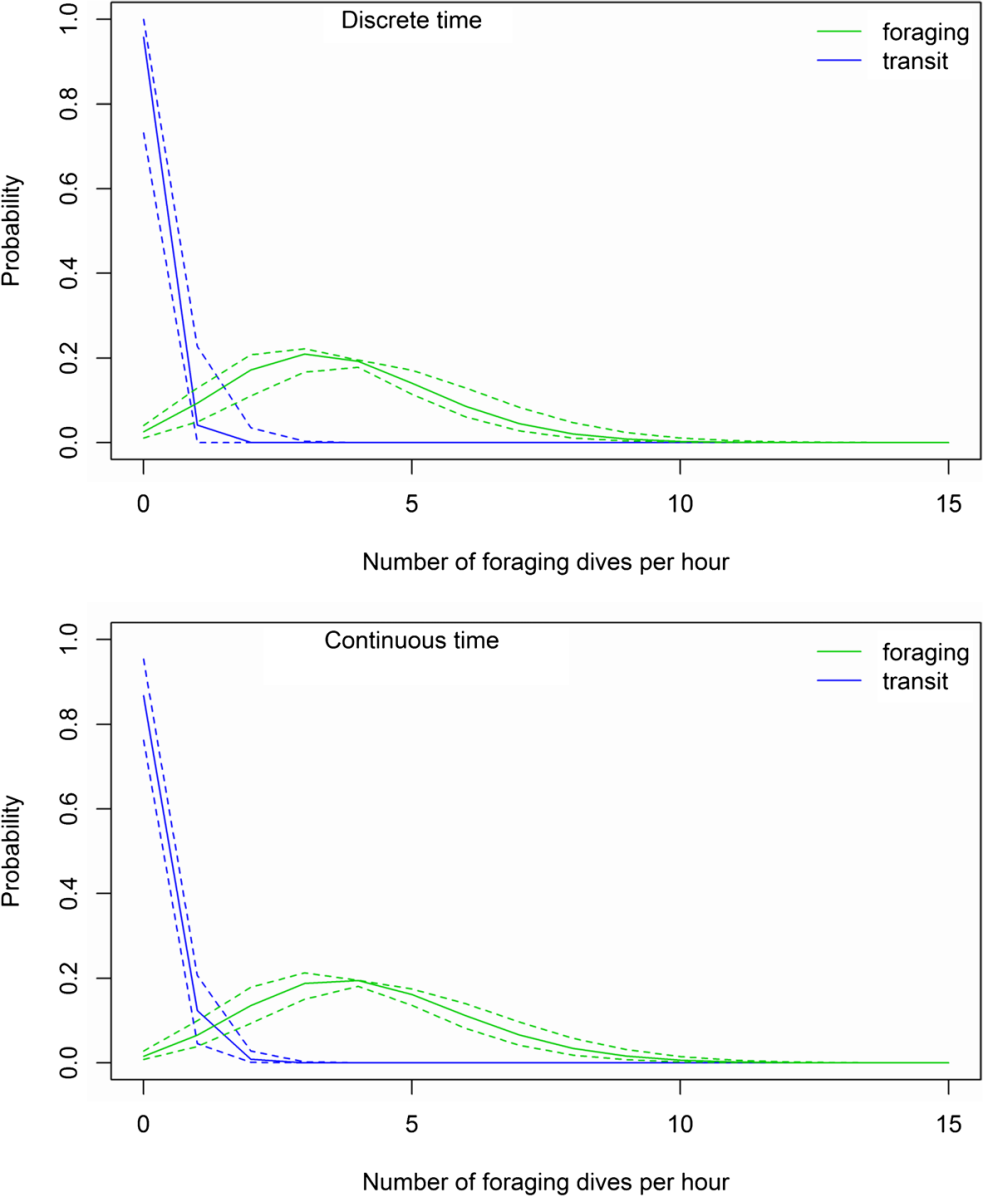
**Hourly probabilities for the number of foraging dives by a northern fur seal while in the foraging and transit states based on discrete- and continuous-time movement process models.** Foraging dives were defined as dives with a max depth >5 m with at least 5 sinuosities (i.e., “wiggles”). Probabilities were calculated from the estimated Poisson distribution for *δ*_*t*_ based on posterior samples for *λ*_*F*_ and *λ*_*T*_. Dashed lines indicate 95% highest posterior density intervals.

Estimated activity budgets to the three movement behavior states were 0.28 (95% HPDI: 0.22-0.37) to resting, 0.36 (0.26-0.39) to foraging, and 0.36 (0.29-0.45) to transit (Figure 2a). Estimated state transition probabilities were ${\widehat{\psi}}_{R,R}$ = 0.81 (0.71-0.92), ${\widehat{\psi}}_{F,F}$. = 0.78 (0.67-0.88), and ${\widehat{\psi}}_{T,T}$ = 0.78 (0.65-0.89), with state-switches more likely to occur between foraging ${\widehat{\psi}}_{F,T}$ = 0.15 (0.05-0.27) and transit ${\widehat{\psi}}_{T,F}$ = 0.14 (0.06-0.22). The bivariate posterior densities for step length and turning angle (Figure 3a) indicate some opportunistic foraging during travelling, with foraging movements often exhibiting high speed and directional persistence typically associated with transit. As expected, time steps with >1 foraging dives were rarely assigned to the transit state (Figure 4a). Also as expected, we found lower speeds and less directional persistence during resting movements and higher speed and more directional persistence during transitory movements.

The estimated error (in meters) for the observation process model was similar between longitude (*σ*_*x*_ = 472; 360 − 596) and latitude (*σ*_*y*_ = 489; 381 − 617) coordinates. Although relatively small, these errors are larger than would typically be expected of GPS location measurement error. We therefore suspect the additional error is attributable to deviations from the simple linear model used to relate the temporally irregular observed locations to temporally-regular predicted locations.

#### Continuous-time formulation

We analyzed the same fur seal data set using a continuous-time model to assess what inferential differences might result by extending the correlated random walk (CRW) models of Jonsen et al. [6] (discrete-time, latent states) and Johnson et al. [8] (continuous-time, state model with known covariates) to a continuous-time CRW model with latent states. The continuous-time correlated random walk (CTCRW) is described by modeling the velocity (instantaneous rate of change) of movement with a bivariate Ornstein-Uhlenbeck (OU) process. The OU process is the continuous-time version of the bivariate autoregressive model Jonsen et al. [6] use to model position difference. The CTCRW locations are then modeled by integrating the velocity process (i.e., the positions are the solution to the stochastic differential equation used to model velocity).

To make the inference comparable between each analysis, we maintained the same hourly structure for the transitions of behavior states. Thus, the models [*Z*_*t*_|**ψ***Z*_*t* − 1_ = *z*] and [*δ*_*t*_|**λ***Z*_*t*_ = *z*] are the same as in the previous discrete-time analysis with the minor technical change that the state *Z*_*t*_ is assumed to be held constant within the interval [*t*, *t* + 1). Also, we use the notation *t*_*i*_ to represent the time of the *i*th observed location in the interval [*t*, *t* + 1).

The CTCRW model is defined by a stochastic differential equation model of velocity ${\boldsymbol{\upnu}}_{t_i}=\left({V}_{x,{t}_i},\ {V}_{y,{t}_i}\right)$ at time *t*_*i*_, such that

3 $$ {V}_{c,{t}_{i+1}}={\gamma}_c+{e}^{-{\beta}_t{\Delta}_{\mathrm{i}}}\left({V}_{c,{t}_i}-{\gamma}_c\right)+\zeta \left({\Delta}_{\mathrm{i}}\right) $$

for each coordinate axis *c* ∈ {*x*, *y*}, where *t* ≤ *t*_*i*_ < *t*_*i* + 1_ ≤ *t* + 1, Δ_i_ = *t*_*i* + 1_ − *t*_*i*_, *γ*_*c*_ is the mean velocity (or drift), *β*_*t*_ is an autocorrelation parameter, $\zeta \left({\Delta}_{\mathrm{i}}\right)=\mathcal{N}\left(0,{\sigma}_t^2\left[1- \exp \left(-2{\beta}_t{\Delta}_{\mathrm{i}}\right)\right]\ /2{\beta}_t\right),$ and *σ*_*t*_ is a parameter controlling the overall variability in velocity. The solution to this autoregressive differential equation is the location ${\boldsymbol{\upmu}}_{t_i}=\left({X}_{t_i},\ {Y}_{t_i}\right)$. Johnson et al. [8] provide details to illustrate that the CTCRW model can be formulated as a linear, Gaussian state-space model that allows efficient calculation of the CTCRW likelihood. For *t* ≤ *t*_*i*_ < *t*_*i* + 1_ ≤ *t* + 1, observation ${\mathbf{y}}_{t_i}=\left({x}_{t_i},\ {y}_{t_i}\right),$ and the vector of the true location and velocity process ${\boldsymbol{\upalpha}}_{t_i}=\left({\boldsymbol{\upmu}}_{t_i},\ {\boldsymbol{\upnu}}_{t_i}\right),$ the state-space model is given by

4 $$ \begin{array}{l}\kern0.5em {\mathbf{y}}_{t_i}={\boldsymbol{\mu}}_{t_i}+{\boldsymbol{\upvarepsilon}}_{t_i}\hfill \\ {}{\boldsymbol{\alpha}}_{t_{i+1}}={\mathbf{T}}_{z,{t}_i}{\boldsymbol{\alpha}}_{t_i}+{\boldsymbol{\eta}}_{z,{t}_i}\hfill \end{array} $$

where $\left[{\boldsymbol{\upvarepsilon}}_{t_i}\right]=\mathcal{N}\left(\mathbf{0},{\tau}^2\mathbf{I}\right)$ and $\left[{\boldsymbol{\eta}}_{z,{t}_i}\right]=\mathcal{N}\left(\mathbf{0},{\mathbf{Q}}_{z,{t}_i}\right)$. The entries of ${\mathbf{T}}_{z,{t}_i}$ and ${\mathbf{Q}}_{z,{t}_i}$ are functions of Δ_i_ and the movement parameters *β*_*t*_ and *σ*_*t*_ (see [8] for details), and as in the discrete-time analysis, the movement parameters depend on the latent state *Z*_*t*_ = *z* via *β*_*t*_ = *β*_*z*_ and *σ*_*t*_ = *σ*_*z*_.

We used an MCMC sampler for Bayesian inference of movement parameters and states. Similar to Johnson et al. [8], we assumed no drift (i.e., *γ*_*c*_ = 0) and similar movement processes in both coordinates (i.e., *β*_*c*,*t*_ = *β*_*t*_ and *σ*_*c*,*t*_ = *σ*_*t*_ for *c* ∈ {*x*, *y*}). The same priors were used for all common variables between the two analyses (e.g., diving rates, behavior states). For the CTCRW movement parameters, we used vague priors on the log scale with the following constraints: *β*_*R*_ > *β*_*F*_ > *β*_*T*_ and *σ*_*R*_ < *σ*_*F*_ < *σ*_*T*_. These constraints imply that movement is typically faster and more correlated as one moves from *R* to *T*. The flat prior [log *τ*] > 10 m was used for the measurement error parameter. The sampler was custom coded in R [47] making use of the FORTRAN coded CTCRW likelihood and posterior track simulation in the R package crawl [48]. The CTCRW likelihood computed via the Kalman filter allowed us to sample from the marginal posterior distribution of the states and movement parameters without having to sample the unobserved ***α***_*t*_ values. The sampled posterior distribution is given by

$$ \left[\boldsymbol{\beta}, \boldsymbol{\sigma}, \tau, \boldsymbol{\lambda}, \boldsymbol{\psi}, \mathbf{Z}\left|\mathbf{x},\mathbf{y},\boldsymbol{\updelta} \right.\right]\propto \left[\boldsymbol{\beta} \right]\left[\boldsymbol{\sigma} \right]\left[\tau \right]{\displaystyle \prod_{t=1}^N\left[{\delta}_t\left|\boldsymbol{\lambda}, {Z}_t\right.\right]\left[{Z}_t\left|\boldsymbol{\psi}, {Z}_{t-1}\right.\right]}\times \kern0.5em {\displaystyle \prod_{t=1}^N{\displaystyle \prod_{i=1}^{k_t}\left[{x}_{t_i},{y}_{t_i}\Big|{Z}_t,{\beta}_t,{\sigma}_t,\tau \right]}} $$

where the right hand-side of the product is the CTCRW likelihood. Note that the true locations $\left({X}_{t_i},\ {Y}_{t_i}\right)$ and velocities $\left({V}_{x,{t}_i},\ {V}_{y,{t}_i}\right)$. have been integrated from the posterior. The benefit of this is that the MCMC sampler for the states and parameters converges more quickly to the approximate posterior distribution. The full algorithm took 66 hours to run (due to coding in R rather than C), however, only 20,000 iterations were necessary to obtain an effective sample of ≥ 4,000 posterior draws. To compare step lengths and turning angles of the CTCRW model to the discrete time model, we needed a sample of hourly locations. To obtain a posterior sample of ***α***_*t*_, *t* = 1, …, *N*, on the hour, the sampling method of Johnson et al. [49] was used at each MCMC iteration as if ***α***_*t*_ was a derived parameter. From the sampled ***α***_*t*_ values, step length and turning angle were calculated for comparison to the equivalent discrete-time quantities.

Estimated activity budgets to the three movement behavior states were 0.10 (95% HPDI: 0.03-0.15) to resting, 0.29 (0.23-0.34) to foraging, and 0.61(0.53-0.67) to transit (Figure 2b). Estimated state transition probabilities were ${\widehat{\psi}}_{R,R}$ = 0.52 (0.10-0.86), ${\widehat{\psi}}_{F,F}$ = 0.75 (0.62-0.86), and ${\widehat{\psi}}_{T,T}$ = 0.82 (0.75-0.89). State-switches to transit were most likely, with ${\widehat{\psi}}_{R,T}$ = 0.40 (0.09-0.81) and ${\widehat{\psi}}_{F,T}$ = 0.23 (0.12-0.35). These are noticeably different from the discrete-time analysis, with much less time spent “resting.” The bivariate posterior densities for step length and turning angle (Figure 3b) also reflect this reduction in state *R*, with more small steps associated with the travel state. However, there were also more large steps associated with the resting state. This calls into question the designation of these states as actually “resting” when using the continuous-time multistate movement model. As in the discrete-time analysis, time steps with >1 foraging dives were rarely assigned to the transit state (Figure 4b). The estimated error (in meters) for the observation process model was $\widehat{\tau}=64$ m (55 m-75 m). Because the observed data linear interpolation does not need to be accounted for, the measurement error variance is noticeably smaller here than in the discrete-time analysis.

Although inferences about time spent foraging were similar between the two approaches, we found considerable differences between the discrete-time and continuous-time formulations with respect to resting and travelling activity. This is counter to the simplistic view that time formulations are merely different means to the same end. The reasons for these differences lie in the underlying relationships of the metrics of movement (speed and directional persistence) that are used to define resting and travelling. Because these metrics are dependent and speed is auto-correlated in the continuous-time model (see **Does a continuous- or discrete-time formulation really matter?**), the lack of auxiliary information (such as metabolic rate) to help distinguish these movement behavior states induces a tendency for the “resting” state to be associated with sudden switches (or change-points) in movement properties during periods with no foraging dives. In other words, instead of identifying periods of slow movement with no foraging dives as intended, the “resting” state serves to break the momentum of the continuous-time movement process.

Although continuous-time formulations necessarily induce dependence between step length and bearing, the differences between our discrete- and continuous-time analyses are not entirely attributable to time formulation *per se*. In order to account for short-term directional persistence in continuous time, Johnson et al. [8] used correlation in the velocity process (Jonsen et al. [6] use the same correlation model in discrete time). Whether in continuous or discrete time, the modelling of velocity clearly induces additional dependence between speed and bearing. Correlated random walk models with two latent movement behavior states can be relatively easy to fit in continuous time (D. Johnson, unpublished data) or when modeling velocity in discrete time [6,50]. However, the modelling of velocity can make it more difficult to characterize and identify >2 distinct movement behavior states with straightforward biological interpretation. While this can be easily avoided in discrete time by modelling step length and bearing independently (as was done here), most continuous-time CRW models are formulated on the velocity process [8,31] (but see [22]).

## Conclusions

Modern tracking and biologging devices allow us to record detailed information on animal location and physiology, thus opening the possibility to better understand the role of movement in population dynamics, animal behavior, and the environment [51,52]. To make the most of these hard-earned data and learn about important aspects of animal movement such as activity budgets, space use, and behavioral responses to landscape features, sophisticated data analysis tools have been proposed. State-space models, where one explicitly accounts for the fact that the observed data arise from a mechanistic or “biological” model that is in turn sampled by an observation model, are currently regarded as the most correct and elegant methods to fit movement models to data [12,52]. We have shown that there exist underappreciated differences among the current available formulations, and although our northern fur seal example focused on state-space models with multiple movement behavior states, our findings have important implications for single-state mechanistic movement process models, including (discrete-time) step-selection or (continuous-time) partial differential equation resource selection models (e.g., see recent reviews by [26,27]).

Although movement is a continuous-time process, it is perhaps more intuitive to think about (and formulate models for) movement in discrete time. In our experience, practitioners find a discrete-time model (Eqs. 1 and 2) and its parameters easier to interpret than its continuous-time counterpart (Eqs. 3 and 4). As we have demonstrated, current discrete-time formulations also provide both flexibility and feasibility for identifying latent behavioral states and incorporating auxiliary biotelemetry or environmental data to inform these states. However, these advantages of discrete-time models do indeed come at a cost. Because inferences from discrete-time models are not time scale-invariant, it is absolutely critical that the chosen time scale between movement steps appropriately matches the animal’s behavioral scales and the frequency of observations.

In addition to loss of resolution, when observations are irregular and/or the frequency of observations greatly exceeds that of the chosen time scale, discrete-time models can suffer from additional lack of fit due to the need to discretize the movement path into temporally-regular locations. This was apparent in the magnitudes of the measurement error terms in our northern fur seal example, where the discrete-time model had larger errors than would normally be expected for GPS data. The need for temporally-regular positions for the entire movement path can also make it more difficult to deal with missing data in a discrete-time framework. While this is less of a problem for terrestrial animals, missing data is a major issue for marine animals due to our inability to obtain locations while underwater.

Continuous time is clearly a more natural representation of movement than discrete time. These models are not dependent on any particular time scale and do not require temporally-regular observations. It is therefore far easier to deal with missing data or changing observational frequencies in continuous time. However, as demonstrated by our northern fur seal example and **Does a continuous- or discrete-time formulation really matter?**, current continuous-time formulations may not be well suited for identifying >2 latent movement behavior states. This is unfortunate because the identification of different behaviors, activity budgets, and how these potentially relate to habitat use and demographic parameters is among the most interesting aspects of movement ecology [51].

Although discrete-time approaches thus far have seen greater development and application, we believe further development of continuous-time models is needed to facilitate more widespread application of these models to real data. For example, the continuous formulations of Blackwell [9], Johnson et al. [8], and Harris and Blackwell [31] could potentially be extended to accommodate “stops” where animals can reorient and change movement state, thereby curbing the momentum inherent to these continuous-time movement process models. By overcoming the hurdles identified here and making latent state-switching models more feasible in continuous time, the best of both worlds may soon be within grasp.

## Abbreviations

CRW: Correlated random walk
CTCRW: Continuous-time correlated random walk
MCMC: Markov chain Monte Carlo
OU: Ornstein-Uhlenbeck
